# Entrainment of Spontaneously Hypertensive Rat Fibroblasts by Temperature Cycles

**DOI:** 10.1371/journal.pone.0077010

**Published:** 2013-10-07

**Authors:** Martin Sládek, Alena Sumová

**Affiliations:** Department of Neurohumoral Regulations, Institute of Physiology Academy of Sciences of the Czech Republic v.v.i., Prague, Czech Republic; Karlsruhe Institute of Technology, Germany

## Abstract

The functional state of the circadian system of spontaneously hypertensive rats (SHR) differs in several characteristics from the functional state of normotensive Wistar rats. Some of these changes might be due to the compromised ability of the central pacemaker to entrain the peripheral clocks. Daily body temperature cycles represent one of the important cues responsible for the integrity of the circadian system, because these cycles are driven by the central pacemaker and are able to entrain the peripheral clocks. This study tested the hypothesis that the aberrant peripheral clock entrainment of SHR results from a compromised peripheral clock sensitivity to the daily temperature cycle resetting. Using cultured Wistar rat and SHR fibroblasts transfected with the circadian luminescence reporter *Bmal1*-dLuc, we demonstrated that two consecutive square-wave temperature cycles with amplitudes of 2.5°C are necessary and sufficient to restart the dampened oscillations and entrain the circadian clocks in both Wistar rat and SHR fibroblasts. We also generated a phase response curve to temperature cycles for fibroblasts of both rat strains. Although some of the data suggested a slight resistance of SHR fibroblasts to temperature entrainment, we concluded that the overall effect it too weak to be responsible for the differences between the SHR and Wistar *in vivo* circadian phenotype.

## Introduction

Circadian rhythms are physiological and behavioral cycles that repeat with a circadian (i.e., about daily) period. They are generated by an endogenous mechanism, anticipate regular changes in environment and adapt the organism to these changes. To fulfill such a role, the circadian rhythms need to be entrained by environmental cues of the solar day. In mammals, light is the dominant entraining cue, but other cues (Zeitgebers), such as temperature, might also be important.

Virtually every cell of the body is genetically equipped to drive a functional molecular clock composed mainly of interconnected clock gene transcriptional and translational feedback loops; for review, see [1]. Protein products of two of these genes (CLOCK and BMAL1) contain the basic helix-loop-helix DNA binding domain and the protein-interaction PAS (PER-ARNT-SIM) domain, form a complex that binds to E-boxes in the promoters of other clock and clock-controlled proteins, such as three Periods (PER1–3) and two Cryptochromes (CRY1,2), and after DNA binding, activate their transcription [2], [3], [4], [5]. After translation, the PER and CRY proteins form complexes that enter the nucleus and inhibit their own transcription, which establishes a negative feedback loop and drives a rhythm [6], [7], [8]. A second feedback loop is formed by two REV-ERB and three ROR proteins, whose expression is activated by E-boxes and the CLOCK-BMAL1 complex, and after translation and translocation to the nucleus, both the REV-ERB and ROR proteins bind to RORE elements in the *Bmal1* promoter and rhythmically switch on and off the expression of *Bmal1* [9], [10]. Several clock genes, such as *Per*, *Rev-Erb*α and *Bmal1*, are thus expressed with a high amplitude circadian rhythm, which can be used to analyze the rhythms *in vitro* and *in vivo*.

The individual clocks must be regularly and mutually synchronized (entrained), or otherwise, they lose their coherence. In mammals, the peripheral clocks are entrained by a master pacemaker in the suprachiasmatic nucleus of the hypothalamus (SCN). The SCN is directly entrained by the light/dark cycle and passes this information to the oscillators in other brain regions and in the peripheral tissues; for review, see [11]. There are likely multiple entraining pathways, and the main entraining factor that carries this information and participates in the resetting of the peripheral clocks has not yet been identified.

Changes in the environmental temperature have been considered as a possible factor to entrain the circadian clocks [12]. The ability to maintain a constant period over a range of temperatures (temperature compensation) has been postulated to be one of the basic circadian clock properties [13]. However, the clock phase may also adapt to the daily temperature cycles [14].

The overt circadian rhythm entrainment by the regular daily environmental ambient temperature changes is important and common among most organisms without thermal homeostasis [15], [16], [17], [18], [19], [20], [21], [22], but previous studies have shown that the ambient temperature cycles can also modestly entrain the locomotor activity in birds [23] and mammals [24], [25], [26], [27], [28]. In mammals, the body temperature is kept within the physiological range, but it varies throughout the day and peaks during the active periods [29]. The variation of temperatures is not only related to the daily activity and rest cycles but is endogenously driven as it persists in conditions when the activity is kept constant, such as in human subjects that maintain a constant routine [30], [31]. Therefore, as one of the central clock output rhythms, daily temperature cycles seem to be a suitable entrainment signal that may easily impinge on most, if not all, of the peripheral oscillators. Importantly, the SCN central pacemaker appears to be considerably resistant to temperature cycles due to its coupled neuronal networks, but it loses its resistance after the disruption of the interneuronal communication by tetrodotoxin [32] or during ontogenesis, before the SCN neuronal network is fully formed [33]. Nevertheless, the temperature entrainment susceptibility of the SCN has yet to be definitively resolved, as several conflicting reports exist [34], [35], [36].

Unlike the SCN, peripheral tissues *in vivo* and cultured cells are much less coupled and represent weak oscillators, as defined by the small radial relaxation rate λ [37], and are generally capable of large phase shifts and period changes during entrainment. Moreover, even temperature cycles with amplitudes within the physiological ranges have been able to sustain the circadian rhythms in otherwise desynchronized clocks in mammalian cell lines and peripheral tissues *in vitro* and *in vivo* [12], [38], [39], [40], [41], [42], [43], [44]. In a recent study, multiple temperature cycles that precisely simulated the physiological daily variations of body temperature were shown to effectively entrain the circadian clocks of mouse cell lines and isolated fibroblasts [45]. This study demonstrated that multiple simulated body temperature cycles of small amplitudes and a wide range of periods were able to act as very efficient entrainment cues.

At the molecular level, the mechanism by which the temperature resets the mammalian circadian clocks seems to be partly mediated by the heat-shock response (HSR) pathway. The HSR pathway serves to protect the cells from temperature extremes, stress- and aging-induced protein-misfolding. The central molecule is the heat shock factor 1 (HSF1), which is activated by environmental stress [46] and binds as a trimeric transcription factor to heat shock elements (HSE) in the promoters of several downstream genes, such as the chaperones *Hsp70* and *Hsp90* [47]. Interestingly, the transcriptional activity of the HSF1 is rhythmic, due to a large fraction of the HSF1 proteins that is translocated to the nucleus during the active phase (i.e., at night in nocturnal mice) when the body temperature is elevated [48]. The HSF1 interferes with the peripheral clock mechanism through the PER2 protein. HSF1 knock-out mice have a longer free-running period than the wildtype mice [49]. Additionally, the pharmacological inhibition [32], [50] or the silencing [45] of HSF1 disables the PER2 induction through a heat pulse and decreases the efficiency of the temperature to entrain the circadian oscillations. However the HSF1 knock-out cells are still able to entrain to temperature cycles, suggesting additional temperature sensors outside the HSF family [45]. The CIRP (cold inducible RNA-binding protein) represents another mechanism by which temperature cycles may regulate the peripheral clock function. The CIRP's rhythmic expression is inversely regulated by temperature cycles but not by the local oscillators [38]. The CIRP binds to hundreds of target mRNAs, including in the untranslated region of the Clock mRNA, and facilitates Clock's translocation from the nucleus to the cytoplasm, which influences the clockwork robustness in a temperature-dependent fashion [51]. The importance of systemic cues such as temperature cycles for the peripheral oscillators is emphasized by the fact that the *Per2* mRNA and several other transcripts remain cyclic even in hepatocytes with a conditionally inhibited local circadian clockwork, because their levels are presumably driven by temperature cycles controlled by a fully functional SCN [52].

Thus far, most studies where the temperature has reset the peripheral oscillators have been performed with mice tissue explants and mouse-derived cell lines. Only a few studies have used rat cell lines [12] or rat SCN explants [33], [34], [35]. In this study, we have focused on the peripheral clock temperature entrainment of spontaneously hypertensive rats (SHR), an animal model of hypertension [53] and metabolic syndrome [54]. In our previous study [55], we have demonstrated that SHR maintained under a light/dark cycle have dampened behavioral output rhythms, an altered expression of clock genes in the colon, suppressed rhythms of various metabolic-related clock-controlled genes and a changed phase relationship between their peripheral tissues. These changes might be due to a decreased ability of the SCN to distribute a robust circadian signal, such as the daily temperature cycle, to fully entrain the output rhythms. Despite some differences in the responses to heat exposure or handling stress, the SHR daily core body temperature profile measured under normal conditions by telemetry does not significantly differ from the control rats' temperature profile [56]. Therefore, this current study was designed to test the hypothesis that the described SHR circadian system anomalies result from a reduced sensitivity to the peripheral clock temperature entrainment. The published SHR genome [57] revealed hundreds of deleted and mutated genes and thousands of genes with single nucleotide polymorphisms; among them were also genes implicated in various signaling cascades involved in the HSR pathway. We used an *in vitro* model of spontaneously immortalized fibroblasts derived from SHR and control rat strain and compared their sensitivity to the temperature changes that reset the circadian rhythms and the entrainment quality. Here, we present evidence that the simple square-wave 24-h periodic temperature cycles of 2.5°C are highly effective Zeitgebers that can phase shift the endogenous clock of immortalized fibroblasts of both rat strains (Wistar and SHR). Although we found small significant differences in the temperature entrainment between SHR and Wistar rat fibroblasts, including differences in their phase response curves, our data as a whole do not support our hypothesis that the disruption in the temperature entrainment is the cause of the SHR circadian system aberrancies.

## Materials and Methods

### Ethics statement

All experiments were approved by Animal Protection Against Cruelty Committee of the Institute of Physiology ASCR, v.v.i., in agreement with Animal Protection Law of the Czech Republic as well as European Community Council directives 86/609/EEC. All efforts were made to minimize the suffering of animals.

### Cell culture and transfection

One adult male SHR and one Wistar rat were killed by decapitation under deep anesthesia (isoflurane). Primary rat fibroblasts were prepared from the skeletal muscle. Minced fragments were incubated 40 minutes in 0.5 mg/ml collagenase (Sigma-Aldrich, USA) in DMEM (D6546, Sigma) with penicillin-streptomycin (1/100, Sigma) and gentamycin (1 µg/ml, Sigma). Digestion was stopped with fetal calf serum (FCS) (Sigma). The suspension was filtered through a 45 µm sifter, washed with a medium and plated in the fresh DMEM containing 20% FCS, 1/100 Glutamax-I CTS (Life technologies, USA) and antibiotics. Fibroblasts emigrating from tissue pieces were allowed to reach 80% confluence, trypsinized, then grown in DMEM (10% FCS, 1/100 Glutamax, antibiotics) and subcultured until presumed spontaneously immortalized cells emerged. The cells were prepared only once, preserved in liquid nitrogen and used for experiments between passages 5–12.

Fibroblasts were plated in 35 mm dishes in DMEM without antibiotics and transfected after reaching 70–80% confluence on the next day by 5 µg Bmal1-dLuc (*Bmal1* promoter in destabilized luciferase-containing plasmid [58], kind gift of M. H. Hastings, MRC-LMB, UK) and 15 µl Genejuice (Merck, Germany). The medium was changed the next day and cells were kept in humidified CO_2_ incubator for 5–15 days. The medium was then changed with a fresh one containing antibiotics every 4–5 days. On the day of the experiment, the medium was replaced with a recording medium that contained 8.3 g/l DMEM without phenol red (cat. no. D5030, Sigma), 4.5 g/l glucose, 1/100 penicillin-streptomycin, 1 µg/ml gentamycin, 0.35 g/l NaHCO_3_, 10 mM HEPES, 1/100 Glutamax, 10% FCS and 0.1 mM luciferin-EF (Promega, USA). Dishes were sealed with glass cover slips and a vacuum grease and then placed into the Lumicycle (Actimetrics, USA) for luminescence recording.

### Real-time bioluminescence recording and analysis

The Lumicycle was placed in an incubator (Memmert, Germany) with programmable square-wave temperature cycles, and the temperature entrainment was performed simultaneously during the luminescence recording. After placing the Petri dishes into the Lumicycle, the cells remained undisturbed (i.e., the cells remained in the incubator, and the medium was not changed). The temperature was set at 36°C constant or to the 12-h temperature cycles of 36°C and 38.5°C. The temperature inside the Lumicycle was recorded in real-time using a GMH3210 digital thermometer (Greisinger Electronic, Germany). Although the higher temperature resulted in higher background luminescence counts, the increase in the noise was only 1–3% of the measured bioluminescence signal. Both Wistar rat and SHR fibroblasts were transfected and recorded simultaneously. The transfections and recordings of the fibroblasts of each rat strain and condition were repeated and assessed in 2–3 independent experiments.

For the quantitative analysis of bioluminescence, the data software package supplied with the Lumicycle was used. For the period and phase calculations, the raw data were baseline-corrected by the 24-h running average. The recording start time in the Lumicycle (i.e., the time of recording medium application) was set as 0 h. A time range of 24 – 96 h was analyzed by fitting a damped sine curve. The resulting period was calculated as the mean ± SD of the 67 individual dishes in the 5 experiments. To measure the period after the temperature cycle entrainment, a damped sine curve was fitted at least 4 peaks after the end of temperature cycles. The differences between the periods and the phase responses of SHR and Wistar rat fibroblasts were evaluated by the unpaired Student *t* test; the differences between the periods before and after the temperature cycle entrainment were evaluated by the paired *t* test with *P*<0.05 required for significance in both cases. The phase shifts were quantified by extrapolating an undamped sine curve (fitted to the times of 12–84 h) from first day after the end of the entrainment. The phase transition curve (PTC) was constructed by plotting the corresponding peak of the extrapolated sine curve (x, old phase) versus the peak of the bioluminescence of the first free-running circadian cycle after the entrainment (y, new phase). The entrainment was verified by fitting and comparing two alternative regression models by the extra sum-of-squares F test: the horizontal straight line (entrainment) and the straight line through the origin with the slope  = 1 (no entrainment, null hypothesis). The calculated phase shift was designated as a phase advance (+) or a phase delay (−) and correlated with the period before the entrainment. The phase response curve (PRC) was constructed by plotting the calculated phase shift versus the approximate original rhythm phase at the start of two-day temperature cycles, with the times 0 and 12 h designated as trough and peak, respectively. The correlations and regressions were performed in Prism 5 (Graphpad, USA).

## Results

### A single 12-h temperature pulse is insufficient to restart the damped circadian oscillations in cultured fibroblasts

To test the needed prerequisites to restart the damped oscillation of Wistar rat and SHR fibroblasts transfected with the *Bmal1*-dLuc reporter, we replaced the growth medium with the recording medium plus 10% serum and immediately placed the fibroblasts into the Lumicycle at a stable 36°C. After recording 7 days, the cell rhythmicity in the majority of the dishes became severely dampened.

**Figure 1:**
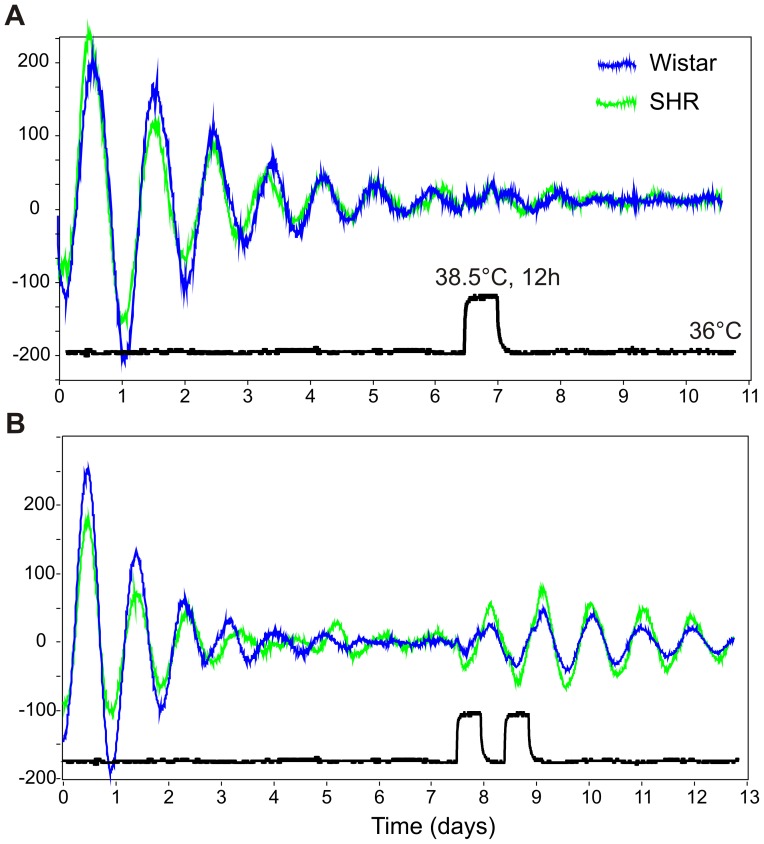
Repeated temperature cycles induce circadian rhythms in damped cell culture oscillations. Cultured Wistar rat (blue) and spontaneously hypertensive rat (SHR; green) primary immortalized fibroblasts were transfected with the *Bmal1*-dLuc reporter and synchronized by medium exchange, and their bioluminescence profiles were recorded in the Lumicycle. The cells were maintained at a constant 36°C until the rhythms damped and were subjected to the temperature stimulus. The temperature readings from the Lumicycle are represented by the black line underneath the bioluminescence traces. **A**: Single 12-h temperature increase from 36°C to 38.5°C, followed by a constant 36°C. **B**: Two consecutive 24-h temperature cycles (2×12 h at 38.5°C/12 h at 36°C).

The administration of a 12-h 38.5°C temperature pulse was not able to restart the oscillations in either Wistar rat or SHR fibroblasts (Fig. 1A). In some cases, when the discernible oscillations still persisted after 7 days, the changes in the phases were not detectable because of a very low oscillation amplitude, which remained unaffected by the temperature stimulus.

Additionally, we tested the efficiency of a short (1-h) high amplitude (43°C) temperature pulse to restart the oscillations (Fig. S1A). We found that in primary fibroblasts, this type of temperature perturbation is more efficient at restarting the *Bmal1*-dLuc oscillations than the long and low amplitude temperature stimulus. No significant differences in sensitivity were observed after both types of temperature stimuli between Wistar rat and SHR fibroblasts.

### Two-day square-wave temperature cycles restart the damped oscillation and set a new phase

Because the single 12-h temperature pulse was insufficient to restart the damped circadian oscillations, we tested the effect of two consecutive square-wave cycles (a constant 36°C, followed by 12 h at 38.5°C, 12 h at 36°C, 12 h at 38.5°C, and a constant 36°C). The temperature readings from the Lumicycle interior during the experiment are plotted in Fig. S1B. The damped circadian oscillations were effectively restarted and persisted for at least 3–4 days after the termination of temperature cycles (Fig. 1B). No discernible differences were detected in the resulting amplitudes and periods of the restarted oscillations between Wistar rat and SHR fibroblasts.

The rhythm period was not significantly affected by the entraining procedure (Fig. S1C). In Wistar rat fibroblasts, the period was 23.1±0.6 h and 23.1±0.5 h before and after the entrainment procedure, respectively (p = 0.93). In SHR fibroblasts, the period was 22.9±0.9 h and 22.8±0.7 h (p = 0.66) before and after the entrainment procedure, respectively. The periods between the rat strains did not differ (p = 0.22), which was consistent with our previous findings [55].

**Figure 2:**
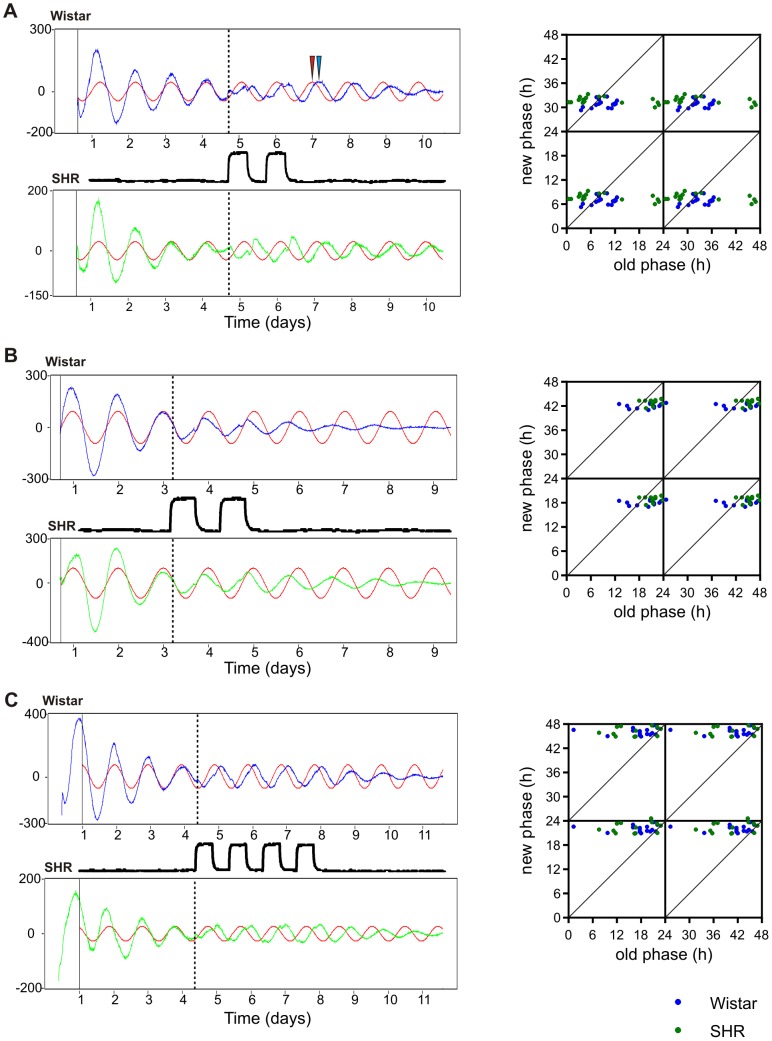
Temperature cycles entrain the fibroblast circadian rhythms. Wistar rat (blue) and SHR (green) fibroblasts were treated as in Fig. 1 and subjected to temperature cycling during the bioluminescence recordings. The temperature readings from the Lumicycle are represented by the black line between the bioluminescence traces. To quantify the phase shift, the free-running circadian rhythm was fitted with a sine curve (red) and extrapolated beyond the time of the entrainment stimulus. The time of the entrainment stimulus administration is depicted by the vertical dotted line. To determine the phase shift, the first peak of the free-running circadian rhythm after the temperature cycle termination (the blue arrow depicts the new phase) was plotted against the extrapolated sine curve peak (the red arrow depicts the old phase). **A**: *Left*, the representative bioluminescence traces of Wistar rat and SHR fibroblast rhythms subjected to two-day temperature cycles (2×12 h at 38.5°C/12 h at 36°C) during the rise of the fifth free-running circadian cycle. *Right*, the PTC of the extrapolated phase before temperature cycles (old phase) plotted against the phase after temperature cycles (new phase) shows significant entrainment to the new phase. The data are double plotted on both axes for clarity. **B**: *Left*, the representative bioluminescence traces of Wistar rat and SHR fibroblast rhythms subjected to two-day temperature cycles during the decline of the third free-running circadian cycle. *Right*, the corresponding PTC shows significant entrainment to the new phase. **C**: *Left*, the representative bioluminescence traces of Wistar rat and SHR fibroblast rhythms subjected to four-day temperature cycles (4×12 h at 38.5°C/12 h at 36°C). *Right*, the corresponding PTC shows significant entrainment to a new common phase for the fibroblasts of both rat strains.

To quantify the phase shifts caused by two square-wave cycles, the fibroblasts were subjected to the entrainment procedure after 3–4 days of free-running circadian rhythms. This entrainment procedure allowed us to accurately assess the oscillation period and construct the phase transition curves well before the oscillations damped. The beginning of two-day temperature cycles is referred to as the entrainment stimulus administration time. It corresponded either with the time of the rise (Fig. 2A) or the fall (Fig. 2B) of the free-running circadian bioluminescence rhythm. The bioluminescence profile from the first 3 days before the entrainment procedure was fitted with a sine curve, which was then further extrapolated. The difference between the first free-running circadian cycle peak after the temperature entrainment (Fig. 2A, blue arrow) and the corresponding peak of the model sine curve (red arrow) were calculated as a phase shift. We constructed the phase transition curves (Fig. 2A, B, right panel; data are double-plotted on both axes for clarity) and analyzed the entrainment efficiency by linear regression. The comparison of the horizontal line model (entrainment) versus the straight line with a slope  = 1 model (no entrainment) revealed a significant entrainment for the fibroblasts from both rat strains (P<0.0001). When the entrainment procedure was applied at the rise of bioluminescence signal, the new phases were clustered around time 6.9±1 h for Wistar and time 7.7±0.9 h for SHR cells (Fig. 2A right; n_Wistar_ = 16, n_SHR_ = 16), and the fibroblasts in the majority of dishes were phase delayed. When the entrainment procedure was applied during the decline of the bioluminescence signal, the new phases were clustered around time 18.0±0.6 h for Wistar rat and time 18.9±0.7 h for SHR cells (Fig. 2B right; n_Wistar_ = 13, n_SHR_ = 12), and the fibroblasts in the majority of dishes were phase advanced. We found small but statistically significant differences in the timing of the phase delays (p = 0.013) and the phase advances (p = 0.003) between the fibroblasts of both rat strains. The direction of the resulting phase shift depended not only on the phase of the entraining stimulus administration but also on the period of the free-running circadian oscillations, which can vary among the fibroblast populations in separate 35-mm dishes. The correlation of the free-running circadian period and the resulting phase shift was highly significant (p<0.0001; data not shown).

We conclude that both Wistar rat and SHR cultured fibroblasts' circadian oscillations can be effectively restarted and efficiently entrained by 2 consecutive square-wave temperature cycles of a moderately low amplitude. We detected small but significant differences in the entrained phase between Wistar rat and SHR fibroblasts; specifically, Wistar rat fibroblasts entrained to an earlier phase than SHR fibroblasts.

### Four-day square-wave temperature cycles cause large phase shifts

Because two-day temperature cycles entrained SHR and Wistar rat fibroblasts to slightly different phases, we examined the hypothesis that a longer entrainment procedure could entrain the fibroblasts of both rat strains to a common phase. Therefore, we applied four consecutive 24-h temperature cycles of the same amplitude as in the previous experiment (Fig. S1D) and analyzed the effect of this entrainment procedure on the period and the phase of the oscillations.

Four-day temperature cycles were applied to Wistar rat and SHR fibroblasts during the same phase of their free-running circadian rhythms. The rhythm period was not significantly affected by the entrainment procedure (Fig. S1E). In Wistar rat fibroblasts, the period was 23.2±0.4 h and 23.0±0.3 h before and after the entrainment procedure, respectively (p = 0.08), and in SHR fibroblasts, the period was 23.5±0.8 h and 22.9±0.8 h before and after the entrainment procedure, respectively (p = 0.08). The phase shifts were quantified as described above (Fig. 2C). The analysis of the PTCs by linear regression showed a significant entrainment of the fibroblasts of both rat strains (P<0.0001). The new phases clustered around time 22.0±0.8 h for Wistar rat cells and time 22.4±1.3 h for SHR fibroblasts (Fig. 2C right; p = 0.41, n_Wistar_ = 16, n_SHR_ = 13) and were not significantly different from each other. Thus, we conclude that four-day temperature cycles efficiently entrained Wistar and SHR fibroblasts to a similar phase.

### The phase response of the temperature entrainment

**Figure 3:**
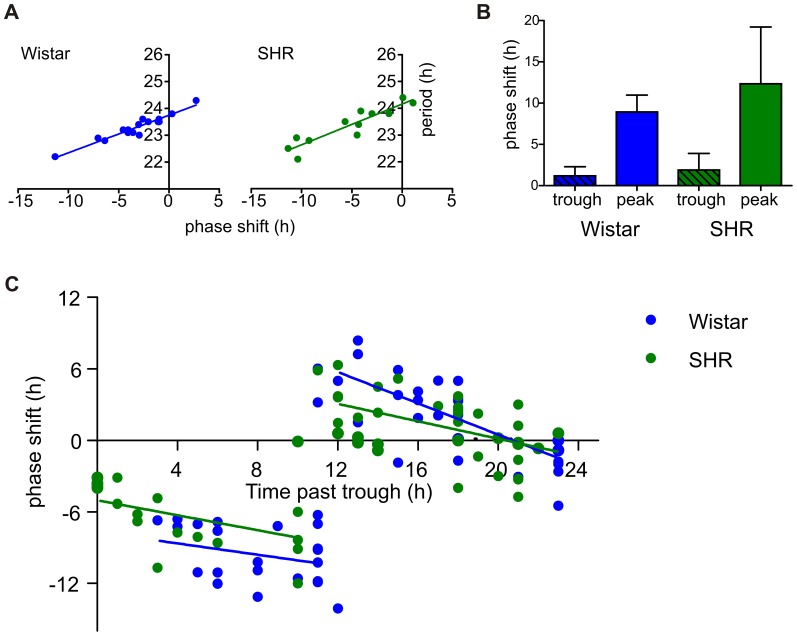
The analysis of the phase response to temperature cycles. **A**: Wistar rat (blue) and SHR (green) fibroblasts were subjected to four-day temperature cycles (see Fig. 2C for details). The phase shift after the entrainment shows a strong correlation with the variable endogenous period of the free-running circadian oscillations of the individual samples. **B**: Wistar rat (blue) and SHR (green) fibroblasts were divided into two groups, which were synchronized by medium exchange at two time points separated by 11.5 h. After three days of recording, both groups were subjected to temperature cycles (2×12 h at 38.5°C/12 h at 36°C) just before their rhythm reached a peak or trough, respectively. The magnitude of the resulting phase shift depends on the oscillation phase at the beginning of the temperature cycle. **C**: The PRC of Wistar rat (blue) and SHR (green) fibroblasts of the two-day temperature cycles (12 h at 38.5°C/12 h at 36°C) constructed from 4 independent experiments. For clarity, the data were separated according to the breakpoint between the phase delays/advances, and the linear portions of the curves were fitted with regression lines.

As expected, the magnitude of the phase shifts caused by temperature cycles was not constant. In our experimental setup, the variation in the periods of the individual samples influenced the actual phase at which the entrainment procedure was applied (i.e., when the first temperature increase of the temperature cycle occurred). The correlation between the initial period and the resulting phase shift (Fig. 3A) was highly significant (p<0.0001).

To examine the phase response of the *Bmal1* circadian expression during square-wave temperature cycles, we prepared Wistar rat and SHR fibroblasts (n = 6 for each group) at two time points, separated by 11.5 h (because the average period of both strains *in vitro* is 23 h). After 3 recording days in a constant temperature, we applied 2 square-wave temperature cycles just before the oscillations reached their peak and trough, respectively (Fig. S1F). After the entrainment procedure, the cells were left free-running at a constant temperature of 36°C. The results demonstrate clear differences between the responses of the oscillations to the imposed temperature cycles that depended on whether the temperature cycle began during the oscillation peak or trough (Figs. 3B and S1F). When the entrainment procedure was applied during the *Bmal1* expression trough, the resulting phase shift was small, and the temperature cycle strengthened the oscillations. When the entrainment procedure was applied during the *Bmal1* expression peak, a more pronounced phase shift occurred and trended towards a reversal of the rhythm after 12 h. No significant differences were detected between the phase shifts of Wistar rat and SHR fibroblasts that were induced by the entrainment procedure at the peak or trough of the oscillations.

Using the pooled data from several independent experiments, we constructed a phase response curve (PRC) for two-day square-wave temperature cycles for both Wistar rat and SHR fibroblasts (Fig. 3C). We plotted the phase shift against the time of the entraining procedure administration. For the analysis, the data were separated according to a breakpoint between phase delays/advances, and linear portions of the curves were fitted with regression lines. The PRC showed a tendency towards increasing phase delays when the entraining stimulus was applied during the rise in the *Bmal1* expression. In contrast, when the entraining cue was applied later during the decline in the *Bmal1* oscillations, the PRC showed a tendency towards decreasing phase advances. For the fibroblasts from both rat strains, the breakpoint corresponded to the time around the rhythm peak. The linear regression showed small differences between the phase response of Wistar rat and SHR fibroblasts. The slopes were the same for both the linear portions of the Wistar rat and SHR fibroblast PRCs (p = 0.77 and p = 0.08, respectively). Small but significant difference was observed between the elevations of the Wistar rat and SHR PRC regression lines plotted in the first part of the PRC (between 0–11 h past the trough, p = 0.03), but not in the second part of the PRC (12–23 h).

In conclusion, the PRCs of the two-day temperature are quite similar for both strains and do not provide enough evidence that phase response to temperature entrainment is compromised in SHR.

## Discussion

In this study, we used 24-h temperature cycles instead of short temperature pulses to study the capacity of the temperature to entrain our circadian clock model. We reasoned that even a very simplified 24-h square-wave temperature cycle may more closely resemble *in vivo* conditions than the short temperature pulse commonly used in the previous studies. A short high amplitude temperature pulse (43°C for 1 h), similar to the pulses used in previous reports [50], [52], was also able to induce oscillations in our model, but the coherence and amplitude of these oscillations were lower than of the oscillations induced by square-wave cycles. One possible reason for lower amplitude of oscillations we observed in our experiment could be an increased sensitivity of our primary immortalized fibroblasts to higher temperatures, which might result in a decrease in cell viability. A single 12-h low amplitude temperature pulse (2.5°C) did not produce any persistent response, and the oscillations remained damped. Similarly, the circadian rhythms of the NIH3T3 fibroblasts have not been induced by a single 12-h temperature pulse but only by a longer (24–60 h) continuous temperature increase to 43°C [40]. In contrast, by repeating the temperature pulse after another 12 h, and thus creating two-day temperature cycles with a period of 24 h, we were able to efficiently restart the damped oscillations of our immortalized rat fibroblasts. Previous reports have shown that the circadian rhythms in mammalian cells can be induced either by high amplitude temperature pulses [50], longer continuous temperature increases [40] or multiple (6-day) temperature cycles [12], [43]. In this study, we demonstrated that, unlike a single 12-h pulse, a second 12-h pulse, which creates two-day temperature cycles and thus defines the 24-h entrainment period, is necessary and sufficient to induce the circadian rhythms in a damped population of rat fibroblasts.

The average free-running circadian period of the fibroblasts was approximately 1 h shorter than the period of the entrainment procedure. While temperature cycles entrained the fibroblasts to a 24-h period through apparent transient cycles, no significant aftereffects on the periods were observed. To assess the phase shifts resulting from temperature cycle entrainment, we extrapolated the original oscillation waveform to predict the phase of the same sample without the temperature entrainment, instead of using a separate control. This approach allowed us to discount the variation in the rhythm parameters, such as the period and amplitude, between the individual samples. These results revealed that in contrast to the period, the phase of the oscillations was significantly affected. Even the shortest temperature cycles were able to induce a permanent phase shift of the circadian oscillations. This observation is in contrast to a previous report [12] demonstrating that RAT1 fibroblast rhythms were sustained but not entrained by a 6-day temperature cycle. However, as discussed elsewhere [45], this difference was most likely due to a different method of rhythm analysis. Indeed, more recent studies using a similar methodology (i.e., a real-time luciferase reporter) showed the ability of 3 or more daily temperature cycles to entrain NIH3T3 fibroblasts, primary *Per2*:luciferase fibroblasts [45], human keratinocytes [44], dermal fibroblasts [42], and pituitary or lung explants [32], [37].

Although our data demonstrate that even the simplest complete daily temperature cycle can efficiently entrain the circadian oscillations of rat fibroblasts, the entrained rhythm amplitude was lower compared with the amplitudes reported by Saini and colleagues [45]. This difference could be attributed to the different model (a stably transfected NIH3T3 cell line) or more likely, to the fact that a well-simulated body temperature cycle, with smooth and continuous temperature changes throughout the daily cycle, was used. Thus, our results indirectly support their hypothesis [45] that the gradual changes in entrainment signal strength, such as the temperature, metabolites or hormones, are physiologically more relevant and that the peripheral oscillators entrain to such cues with a higher efficiency. In parallel, the photoperiodic entrainment of the suprachiasmatic nuclei via a gradual "twilight" provided a stronger clock entrainment in the neuronal subpopulations than the abrupt light-dark transitions normally used in laboratory conditions [59]. We hypothesize that the lower efficiency of the abrupt temperature changes to entrain the fibroblast clock might be due to presence of several differently phased cell populations; some cells are already entrained to the new phase determined by temperature cycles, and other cells are still locked to the old phase determined by the original medium exchange. These individual out-of-phase circadian rhythms may lead to a reduction in the resulting output rhythm amplitude of the cell population. Indeed, such differently phased cell subpopulations during the temperature cycle entrainment have been identified by single-cell oscillation recordings by a luminescence microscope [45].

No significant differences were observed between the Wistar rat and SHR fibroblast average clock periods measured after the temperature entrainment, although the experimental variation between the individual samples was high. These results are in agreement with our previous report [55] demonstrating no detectable differences in the periods of Wistar rat and SHR fibroblasts after a pharmacological reset of the clocks. Although we detected a trend for SHR fibroblasts to entrain towards a later phase than Wistar cells, we considered the overall effect, though statistically significant, not large enough to cause any effect *in vivo*. The administration of a longer lasting and thus stronger entrainment stimulus, such as four-day temperature cycles, eliminated the significant difference between the Wistar rat and SHR phases, and the fibroblasts of both rat strains entrained similarly.

For a more detailed comparison of the sensitivities of Wistar rat and SHR fibroblasts to temperature entrainment, we generated a multiple pulse phase response curve to two-day temperature cycle. Previously, the PRCs have been mostly constructed to a single short entraining cue; however, in *in vivo* experiments, the PRCs developed from multiple pulses, such as 3-day light cycles [60] or 3 consecutive days of exogenous melatonin administration [61], have also been used. To the best of our knowledge, this study described for the first time the PRC for the temperature entrainment of cells. Previously, the PRCs were generated either for different models, such as peripheral tissue [32] or SCN [35] explants, or to different Zeitgebers, such as glucocorticoid signaling [62], or with a combination of both [63]. The PRC waveform obtained in our model resembled the PRC waveform reported by Buhr and colleagues [32] for the temperature entrainment of cultured lung and pituitary explants from *Per2*:LUC knock-in mice. There was a difference in the position of the breakpoint between these PRCs. Whereas the breakpoint position was located 10–12 h past the trough of the free-running rhythm in our model, it occurred approximately 3 h past the trough in the tissue explant model (40). This might be because *Bmal1*-dLuc and *Per2*:LUC reporters are rhythmically expressed in a phase angle of 8–10 h, which is similar as their wild-type counterparts (*Bmal1* mRNA and PER2 protein) both *in vivo* [8], [9] and *in vitro* [45], [64]. While the *Per2*:LUC bioluminescence is directly responsive to temperature change due to the presence of heat shock elements in the *Per2* promoter, the *Bmal1* promoter-driven luciferase reporter [58] is influenced by the temperature only indirectly [45]. The discrepancies in both PRCs could also reflect the different strength of the used Zeitgeber (two-day temperature cycles vs. single 1-h temperature pulse). Overall, the phase responses of Wistar and SHR fibroblasts were of similar magnitudes and also the shape of both PRCs did not largely differ.

In our experimental setup, a relatively small 1 h difference in period between the individual cell cultures could result in a phase shift of up to 7 h (Fig. 3A). This occurred because the phase of the individual cultures at the start of the entrainment stimulus varied as the initially small period difference accumulated over the course of 4 days. In cases, when the beginning of the temperature cycle coincided with the peak of the rhythm (i.e. around the PRC breakpoint), the resulting phase shift was large. The period and resulting phase shift were highly correlated for both strains.

## Conclusion

We report that even simple 24-h square-wave periodic temperature cycles with amplitudes of 2.5°C are highly effective Zeitgebers that can phase shift the endogenous clock of immortalized skeletal muscle fibroblasts. The phase shift magnitude and the phase angle depends on the endogenous period of the free-running fibroblast clock and on the relative strength of the entrainment stimulus as determined by the completeness and number of temperature cycles. Whereas a single 12-h temperature pulse fails to restart the rhythmic circadian expression of the *Bmal1* gene, two-day temperature cycles can efficiently restart the damped oscillations. This study is the first to report the PRC to temperature cycles for cultured cells. Despite some minor differences, both genotypes were capable of robust phase resetting via temperature cycles. We conclude that the differences in SHR and Wistar circadian system could not be attributed to varied sensitivity of peripheral oscillators to temperature entrainment.

## Supporting Information

Figure S1 **Supplementary data.** **A**: Wistar rat (blue) and SHR (green) fibroblasts were treated as in Fig. 1, maintained at a constant 36°C until the rhythms damped and then subjected to a single 1-h temperature pulse of 43°C, depicted by the red arrow. **B**: The temperature readings from the Lumicycle apparatus (the thermometer probe was placed 10 cm above the Petri dish) during the experiments employing two-day temperature cycles. **C**: The period of Wistar rat (left) and SHR (right) fibroblasts before the start (old period) and after the end (new period) of the temperature entrainment from two-day temperature cycles. **D**: The temperature readings from the Lumicycle during the experiments employing four-day temperature cycles. **E**: The period of Wistar rat and SHR fibroblasts before the start and after the end of the temperature entrainment from four-day temperature cycles. **F**: Wistar rat (left) and SHR (right) fibroblasts were divided into two groups, which were synchronized by medium exchange at two different time points separated by 11.5 h. After three days of recording, both groups of fibroblasts were subjected to temperature cycles (2×12 h at 38.5°C/12 h at 36°C, depicted by the black line beneath the traces) just before their rhythm reached the peak (red traces) or trough (black traces), respectively. See Fig. 3B for the results.
(TIF)
