# Supplementary figures and images for: Entrainment of Spontaneously Hypertensive Rat Fibroblasts by Temperature Cycles
Source: PLoS One. 2013 Oct 7;8(10):e77010. doi: 10.1371/journal.pone.0077010 (PMC3792033; doi:10.1371/journal.pone.0077010)

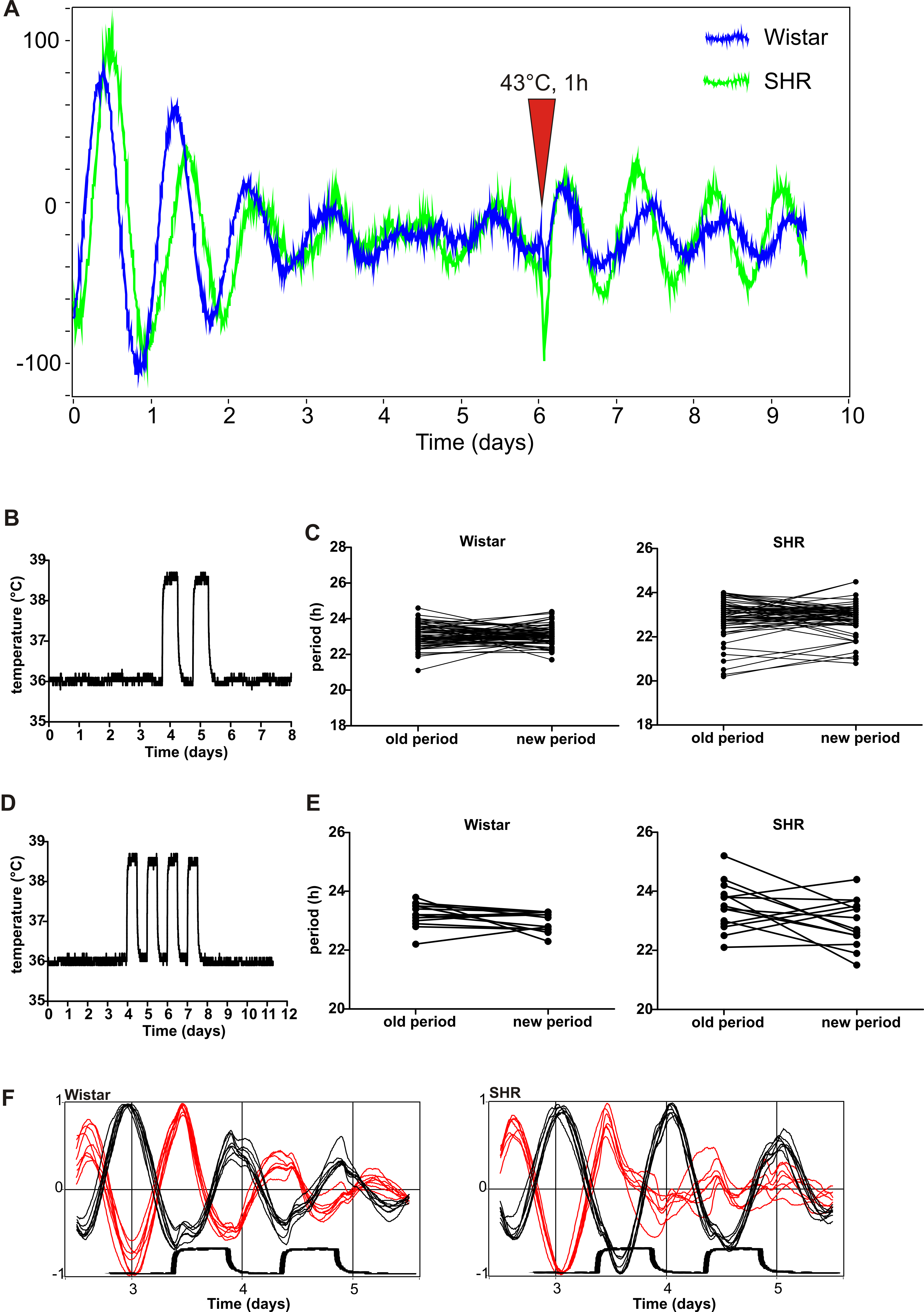

Supplement: Figure S1 — Supplementary data. A: Wistar rat (blue) and SHR (green) fibroblasts were treated as in Fig. 1, maintained at a constant 36°C until the rhythms damped and then subjected to a single 1-h temperature pulse of 43°C, depicted by the red arrow. B: The temperature readings from the Lumicycle apparatus (the thermometer probe was placed 10 cm above the Petri dish) during the experiments employing two-day temperature cycles. C: The period of Wistar rat (left) and SHR (right) fibroblasts before the start (old period) and after the end (new period) of the temperature entrainment from two-day temperature cycles. D: The temperature readings from the Lumicycle during the experiments employing four-day temperature cycles. E: The period of Wistar rat and SHR fibroblasts before the start and after the end of the temperature entrainment from four-day temperature cycles. F: Wistar rat (left) and SHR (right) fibroblasts were divided into two groups, which were synchronized by medium exchange at two different time points separated by 11.5 h. After three days of recording, both groups of fibroblasts were subjected to temperature cycles (2×12 h at 38.5°C/12 h at 36°C, depicted by the black line beneath the traces) just before their rhythm reached the peak (red traces) or trough (black traces), respectively. See Fig. 3B for the results. (TIF) [file pone.0077010.s001.tif]
